# Perceived Performance, Intrinsic Motivation and Adherence in Athletes

**DOI:** 10.3390/ijerph17249441

**Published:** 2020-12-16

**Authors:** Bartolomé J. Almagro, Pedro Sáenz-López, Sebastián Fierro-Suero, Cristina Conde

**Affiliations:** Faculty of Education, Psychology and Sport Sciences, Universidad de Huelva, Avda. Tres de marzo s/n, 21071 Huelva, Spain; almagro@dempc.uhu.es (B.J.A.); cristina.conde@dempc.uhu.es (C.C.)

**Keywords:** self-determination, psychological needs, sport, performance, psychometric properties

## Abstract

Sports performance depends on a complex interaction of variables, such as psychological, physical, technical and tactical abilities. The purpose of the three studies described in this article was to validate an instrument to measure perceived performance in athletes (studies 1 and 2), and to analyze the predictive power of perceived performance, basic psychological needs and intrinsic motivation on the intention to be physically active (which was used as a predictor of adherence to sport) (study 3). In study 1, the Perceived Performance in Sports Questionnaire was validated in the Spanish sports context, analyzing its factorial structure, reliability, and temporal stability with 146 soccer players. The objective of study 2 was to check the factorial structure of the questionnaire with an objective performance measure (points scored and time played). The sample was 180 young basketball players. The objective of study 3 was to analyze the predictive power of basic psychological needs in exercise, intrinsic motivation, and perceived performance on the intention to be physically active in 339 athletes of fifteen different sports. The results show the reliability and validity of the questionnaire, and positive correlations with the points scored. Finally, the analysis of the structural equation model showed that the satisfaction of the need for competence predicted perceived performance and intrinsic motivation predicted perceived performance and intention to remain physically active.

## 1. Introduction

### 1.1. Perceived Performance in Sport

Sports performance depends on a complex interaction of variables [1]. Every sportsperson wants to perform well, to win, and to improve their performance [2,3]. All players try their best to achieve individual and, if applicable, team objectives. Sports success requires that athletes, within certain limits, perform to the best of their ability, for the most successes [2].

The study of sports performance has focused on various variables [4]. Psychological abilities have a significant influence on performance [5]. The role of the player’s psychological characteristics is relevant not only because of its direct impact on the athlete’s performance, but also as a mediator between the athlete’s physical, technical, and tactical skills and performance in competition [6,7,8,9,10]. The study of these variables can help athletes to optimize their performance [11].

In young people, perception of their performance could be as important as the performance because they need to feel committed and motivated as part of the team [1]. They feel part of the team if they think that they are important to the team´s performance. Thus, it would be useful to have a tool that evaluates the perception of performance and that can help to know its relationship with other variables as the motivation.

### 1.2. Performance and Intrinsic Motivation in Sport

Various studies have shown motivation as a psychological factor related to sports performance [12,13,14,15,16]. In the framework of the self-determination theory (SDT), motivation varies over a continuum that ranges from amotivation, extrinsic motivation to intrinsic motivation [17,18]. The state of amotivation is characterized by a lack of intention to participate. Extrinsic motivation is divided into four types of regulation. The first two, external and introjected regulation, are considered controlled regulatory types, which means that behavior is regulated by non-self-determined processes. The next two, identified and integrated regulation, are considered autonomous regulatory types, which means behavior is regulated by internal self-determined forces. Finally, intrinsic motivation is the most self-determined form and occurs when the athlete takes part due to an interest in, or enjoyment of, the activity itself [19]. The most self-determined forms of motivation are related to positive behavioral consequences such as engagement, well-being, or healthy and mature relationships [20]. In several studies that used the SDT theoretical framework in the sports context, one of these consequences was improved performance of the athletes [21,22,23]. Intrinsic motivation improves performance even when the athletes still have the same level of competence [2].

According to this theory, prediction of self-determined motivation requires satisfaction of the three basic psychological needs: competence, autonomy, and relatedness [20]. Competence refers to basic need to feel effectance and mastery. Autonomy is the need to self-regulate one’s experiences and actions. Relatedness concerns feeling socially connected [20,24]. The more the athletes’ basic needs are satisfied, the more their levels of self-determined motivation may increase, leading to enhanced psychological functioning. Thus, if athletes feel their three basic psychological needs are satisfied, their performance could improve. In fact, some studies have linked the satisfaction of basic psychological needs with performance in sport [25], especially for the case of the need for competence [26].

### 1.3. Performance and Intention to Be Physically Active

Physical activity has many benefits for young people [27]; for instance, in physical health (preventing obesity, cardiovascular disease or musculoskeletal injuries), in psychological health (self-concept, autonomy, anxiety or depression) and even in social integration (for the transmission of knowledge, values and norms) [28,29,30]. For this reason, understanding why young people adhere to physical activities has attracted attention in sports science research [31,32,33,34]. This research focused on young athletes (between 13 and 18 years old), because various studies reported that the highest number of dropouts in sports occur during this period [35,36,37]. Different factors (lack of enjoyment, perceptions of competence, social pressures, competing priorities and injuries) affect the sports dropouts of young athletes, highlighting the lack of competence at the time of the decision [35,38], which can cause poor performance [39]. In this sense, numerous studies show that coaches have a great influence on athletes [33,40,41]. In fact, the motivational model of the coach–athlete relationship describes how coaches may influence athletes’ motivation, meaning that they are important determinants of performance and persistence [42]. Therefore, the intention expressed by children to practice sport can be a good indicator of the motivation towards this sporting activity as well as a strong predictor of this behavior [31]. Along these lines, positive experiences when practicing sports may contribute to a greater intention to continue involvement in physical activities [43] and thus generate adherence to sports practice [34]. Moreover, these positive experiences are related to an adequate self-concept as well as a high perception of competence when practicing a sport [27]. Hence, it is necessary to know how the perception of performance relates to the intention to be physically active in sports.

The objective of this research was to provide further evidence that the relations between perceived performance, motivation, and the intention to be physically active in young athletes. For this, three studies were carried out, two focused on validating an instrument to measure perceived performance in athletes (studies 1 and 2), and another that focused on analyzing the relations between basic psychological needs, intrinsic motivation, perceived performance, and the intention to continue being physically active in athletes. (study 3). Thus, the following hypotheses were presented: (1) the Perceived Performance in Sports Questionnaire will present adequate psychometric properties; (2) the basic psychological needs will predict the intrinsic motivation and the perceived performance, which, in turn, will predict the intention to being physically active.

## 2. Study 1

The purpose of this study was to adapt the Perceived Performance in Sport Questionnaire (Questionário de Perceção do Rendimento no Desporto, (QPRD) in Portuguese) [44] to the Spanish sports context. For this, its factorial structure, reliability, and temporal stability were analyzed.

### 2.1. Methods

#### 2.1.1. Participants

There were 146 (131 boys and 15 girls) participants, young soccer players aged between 14 and 35 years old (M = 20.50; SD = 4.11) from four Spanish cities. All of them competed every weekend with their soccer teams. These soccer players train an average of 6.13 h a week (SD = 2.41). The selection of the sample was done according to a non-probabilistic or convenience sampling.

#### 2.1.2. Measures

##### Perceived Performance

An adaptation in Spanish of the Perceived Performance in Sports Questionnaire (Questionário de Perceção do Rendimento no Desporto, QPRD) was used [44]. This instrument is made up of 5 elements (for example, “I consider my performance is good”), which are unique factors in evaluating the perception of athletes’ performance of their sports activity. The responses to the items are given on a Likert-type scale from 1 (Strongly disagree) to 5 (Strongly agree). The previous sentence was: “Overall, during the competition: …”.

#### 2.1.3. Procedure

This study was approved by the Andalusian Biomedical Research Ethics Committee (code: PIERP2020) and was carried out in accordance with the ethical principles of the American Psychological Association [45].

Participation in the study was solicited through direct contact with soccer clubs and coaches. Moreover, all participants provided written consent and, if they were under-aged, their parents or legal guardians also gave consent for their children to participate in the study.

As it was a questionnaire previously validated with Portuguese athletes, a circular translation of the items was performed. For this purpose, first, two translators translated the scale into Spanish; later, two other translators translated it back into the original language. After that, the original and final versions were compared to check that both scales had the same meaning.

The administration of the final questionnaire (Appendix A, Table A1) was done in the presence of a study researcher, to briefly explain how to fill in the instrument, solving any doubts that might arise. Anonymity and honesty in the responses were insisted upon. The approximate completion time was five minutes.

#### 2.1.4. Data Analysis

The psychometric properties of the Perceived Performance in Sports Questionnaire were analyzed. An exploratory factor analysis (EFA) was used to check if the five items were grouped into a single factor. The reliability of the scale was evaluated using Cronbach’s alpha. Temporal stability was also evaluated through test-retest.

### 2.2. Results

#### 2.2.1. Exploratory Factor Analysis

Before performing the exploratory factor analysis, the Kaiser–Meyer–Olkin test (KMO = 0.81) and Bartlett’s statistic indices (χ^2^ = 309.05, *p* < 0.001)) were calculated. These results show the suitability of the data for the analysis. Exploratory factor analysis (EFA) with the maximum likelihood method was employed to identify the latent dimensions that underlie the data. In the exploratory factor analysis with the maximum likelihood method, an eigenvalue of 3.12 and an explained variance of 62.3% were obtained (see Table 1). The results of the EFA show that the five items were grouped into a single factor: perceived performance.

#### 2.2.2. Reliability and Temporary Stability

The internal consistency analysis of the instrument was performed by calculating Cronbach’s alpha coefficient, whose value was 0.84.

Temporal stability was measured by performing test-retest. The Perceived Performance in Sports questionnaire was administered to the 146 young soccer players with whom the study was conducted. Thirty days After the first questionnaire, the retest was carried out with 21 young people from the sample. After analyzing the corresponding data, a correlation coefficient of 0.72 was found.

## 3. Study 2

The objective of this study was to confirm the factorial structure of the questionnaire obtained in Study 1 with an independent sample. In addition to checking the validity of the criterion, the relationship between perceived performance and a more objective performance measure was verified.

### 3.1. Methods

#### 3.1.1. Participants

Two samples of young basketball players were used to carry out Study 2. One of the samples, comprising 130 basketball players (82 males and 48 females) aged 14 to 22 (M = 15.85; SD = 1.65), was used to test the factorial structure of the questionnaire through a confirmatory factor analysis. These basketball players belonged to various clubs in the provinces of Huelva and Sevilla. They competed every weekend with their basketball teams. Furthermore, these players train an average of 5.23 h per week (SD = 1.76). The other sample consisted of 50 (24 boys and 26 girls) young basketball players aged between 12 and 16 years (M = 14.22; SD = 1.04) from Huelva. These players were evaluated to compare their perceived performance to the real performance through the number of points scored and minutes played in the last 3 games. The selection of both samples was made according to convenience sampling.

#### 3.1.2. Measures

##### Perceived Performance

The version obtained from the Perceived Performance in Sport Questionnaire (Appendix A, Table A1) was used after the exploratory factor analysis performed in Study 1. Cronbach’s alpha obtained for this second study was 0.94.

##### Points Scored in the Last Three Matches

As a more objective measure of the individual performance of the basketball players, the variable points scored in the last three basketball games was used. For this, the official records of those matches were used. In several studies, points scored were used as part of an individual’s performance in basketball [46,47,48].

##### Playing Time of Each Player in the Last Three Matches

Another measure that can often be linked to the performance of the basketball player is playing time in games. In this case, the playing time in the last three matches for each player was used. Some studies used playing time as another indicator of basketball player performance [47,49].

#### 3.1.3. Procedure

The process of collecting the data and the instructions given on completing the questionnaire were similar to those described in Study 1.

#### 3.1.4. Data Analysis

First, with the sample of 130 basketball players, a confirmatory factor analysis (CFA) was performed. The currently most recommended adjustment indices were used to evaluate the proposed models: χ^2^/d.f., Comparative Fit Index (CFI), Tucker Lewis Index (TLI), Incremental Fit Index (IFI), Root Mean Square Error of Approximation (RMSEA) and Standardized Root Mean Square Residual (SRMR). Values equal to or greater than 0.90 in CFI, TLI and IFI, lower than 5 for χ^2^/d.f., and lower than 0.08 for RMSEA and SRMR are considered acceptable fit indices [50]. Moreover, the Pearson correlation coefficient was calculated between perceived performance, points scored and playing time.

### 3.2. Results

#### 3.2.1. Confirmatory Factor Analysis

**Figure 1:**
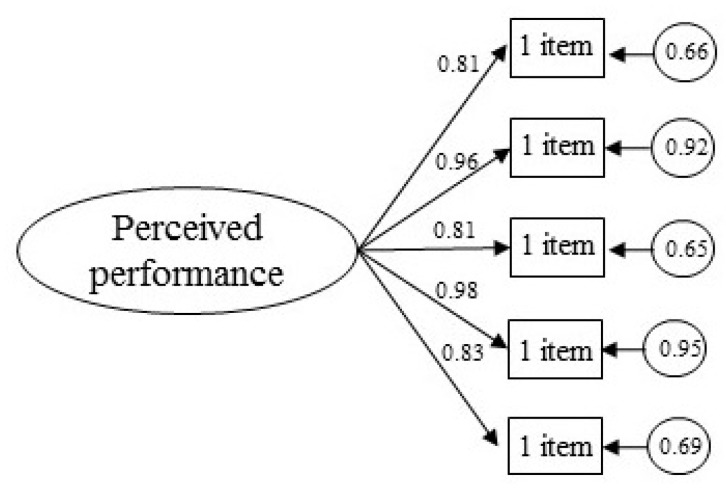
Confirmatory factor analysis of the Perceived Performance in Sports Questionnaire.

The maximum likelihood estimation method was used in the CFA to examine the single-factor structure of the model. The results of the analysis show a good fit to the single-factor model: χ²/d.f. = 4.9, *p* < 0.001, CFI = 0.97, TLI = 0.94, IFI = 0.97, RMSEA = 0.07, SRMR = 0.03. The standardized factor loadings were statistically significant (*p* < 0.001) and ranged from 0.81 to 0.98 (Figure 1).

#### 3.2.2. Criteria Validity Analysis: Bivariate Correlations

To test the relationship of the perceived performance with other variables, a Pearson correlation analysis was performed. As can be seen in Table 2, positive and statistically significant correlations were found between perceived performance and the points scored. The points scored in the last three games correlated positively and significantly with the player’s playing time.

## 4. Study 3

The objective of Study 3 was to analyze the predictive power of basic psychological needs, intrinsic motivation and perceived performance on athletes’ intention to be physically active.

### 4.1. Methods

#### 4.1.1. Participants

Study 3’s sample consisted of a total of 339 athletes, of whom 139 were girls and 200 were boys, whose ages were between 12 and 28 years (M = 17.69, SD = 4.06). All the participants practiced some competitive sport in the provinces of Huelva or Sevilla. The sample was collected from both municipal sports schools and sports clubs and from various sports: basketball (*n* = 83), soccer (*n* = 79), futsal (*n* = 35), handball (*n* = 23), volleyball (*n* = 21), paddle tennis (*n* = 20), athletics (*n* = 15), indoor hockey (*n* = 12), swimming (*n* = 10), karate (*n* = 9), cycling (*n* = 8), badminton (*n* = 8), table tennis (*n* = 6), triathlon (*n* = 6) and tennis (*n* = 4). These athletes trained for an average of 5.39 h (SD = 3.52). The selection of the participants was done using convenience sampling.

#### 4.1.2. Measures

##### Basic Psychological Needs

The Spanish version [51] of the Basic Psychological Needs in Exercise Scale (BPNES) [52] was used, adapted to a sports context [53]. The scale included 12 items covering the three needs for competence (example: “I have had great progression with respect to the desired result”), autonomy (example: “The training program that I still fit my interests”), and relatedness (example: “I feel very comfortable when I exercise with the other athletes”). The questionnaire was administered with the instructions, “During the training…”. A Likert scale was used, ranging from 1 (Not true at all) to 5 (Very true). Cronbach’s alpha values of 0.73 for competence, of 0.71 for autonomy and 0.75 for the relatedness were obtained.

##### Intrinsic Motivation

The intrinsic regulation factor of the Spanish version [54] of the Behavioral Regulation in Sport Questionnaire (BRSQ) [19] was used. The intrinsic regulation factor measures intrinsic motivation and is made up of 4 items (example: “because I enjoy it”). The answers were responded to with a Likert scale that ranged from 1 (Strongly disagree) to 7 (Strongly agree). Cronbach’s alpha was 0.89.

##### Perceived Performance

The validated version of the Perceived Performance in Sport Questionnaire was used in Studies 1 and 2. Cronbach’s alpha obtained in this study was 0.87.

##### Intention to be Physically Active

The Spanish version [27] of the Measure of Intention to be Physically Active [55] was used. It consists of five items for measuring the subject’s intention of being physically active (for example, “I am interested in developing my physical fitness”). The items are preceded by the phrase “Regarding your intention to practice sport …”. The answers were responded to with a Likert scale ranging from 1 (Strongly disagree) to 5 (Strongly agree). The analysis of the internal consistency revealed a Cronbach’s alpha of 0.77.

#### 4.1.3. Procedure

The process of collecting the data and the instructions given to complete the questionnaire were similar to those described in Study 1. The main difference is that the athletes took about 10 min to fill out the questionnaire, since they had to answer more items.

#### 4.1.4. Data Analysis

The descriptive statistics of the various variables of the study and the bivariate Pearson correlations were calculated. Next, a structural equations model was done to analyze the hypothesized relations between the variables. The various analyses were carried out with the SPSS 26.0 and AMOS 26.0 statistical packages.

### 4.2. Results

#### 4.2.1. Descriptive and Bivariate Correlation Analyses

Table 3 gives the descriptive statistics (means and standard deviations) of each of the study’s variables and the bivariate Pearson correlations.

#### 4.2.2. Structural Equations Model

**Figure 2:**
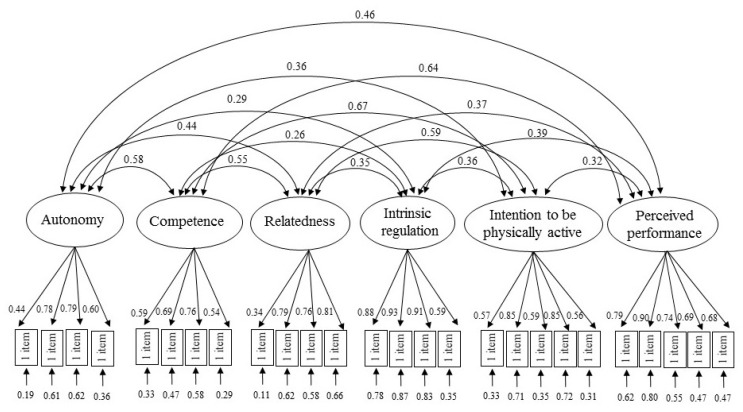
Measurement model.

In order to test the structural equations model (SEM) presented later, a measurement model was first carried out (Figure 2), which allowed for construction of a validity for the scales and corresponded to a confirmatory factorial analysis (CFA), based on the 26 observed measurements and on the six latent constructs. To verify the validity of the measurement model, the following goodness-of-fit indices were taken into account: the ratio between chi-squared and degrees of freedom (χ^2^/d.f.), the Comparative Fit Index (CFI), the Incremental Fit Index (IFI), Tucker Lewis Index (TLI), the Standardized Root Mean Square Residual (SRMR), and Root Mean Square Error of Approximation (RMSEA). In this respect, the indices of the measurement model were appropriate: χ^2^ (290, N = 339) = 513.12, p = 0.00, χ^2^/d.f. = 1.81, IFI = 0.95, TLI = 0.94 CFI = 0.95, SRMR = 0.05, RMSEA = 0.05.

The second step of the method was to analyze the existing predictive relations between the variables of the study through a structural model. The model hypothesized that the basic psychological needs of autonomy, competence, and relatedness with others would positively predict intrinsic motivation. Furthermore, intrinsic motivation would positively predict perceived performance. It was expected that the intention to be physically active would be positively related to the perceived performance in sport and intrinsic motivation. However, this model did not show adequate fit indices: χ^2^ (290, *N* = 339) = 652.84, *p* = 0.00, χ^2^/d.f. = 2.25, IFI = 0.91, TLI = 0.90, CFI = 0.91, SRMR = 0.06, RMSEA = 0.10. For this, to achieve better model fits, a direct relationship was added between perceived performance and the intention to be physically active. Furthermore, the relationship between the need for competence and intrinsic motivation was eliminated, since this relationship was not statistically significant. After these changes, the indices of the measurement model were appropriate: χ^2^ (292, *N* = 339) = 636.34, *p* = 0.00, χ^2^/d.f. = 2.19, IFI = 0.92, TLI = 0.91, CFI = 0.92, SRMR = 0.06, RMSEA = 0.08.

**Figure 3:**
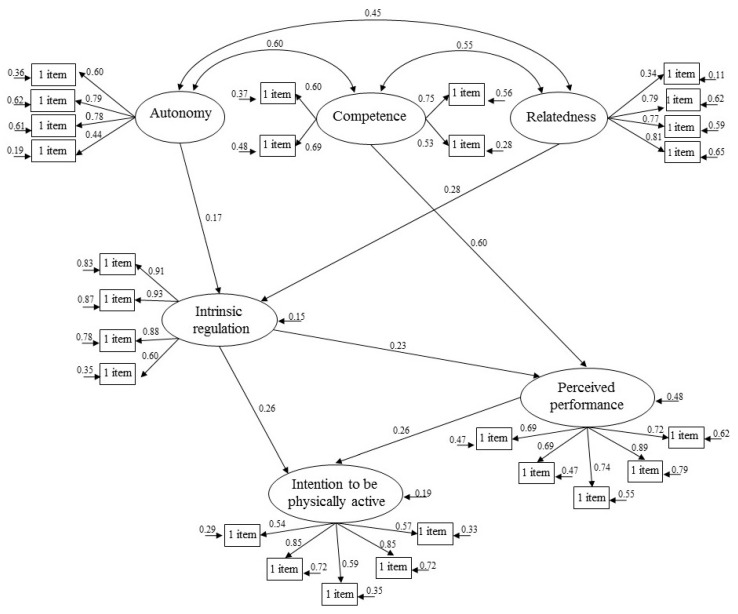
Structural equations model.

As can be seen in Figure 3, the results of the analysis of the structural equation model showed that the satisfaction of the needs for autonomy and relatedness with others would positively predict intrinsic motivation. The need for competence predicted perceived performance. For its part, intrinsic motivation predicted perceived performance and intention to remain physically active. Finally, it was also found that perceived performance predicted the athlete’s intention to be physically active in the future, showing the direct and indirect effect of intrinsic motivation on the intention to be physically active.

## 5. Discussion

This study was designed to validate the Spanish version of the Perceived Performance in Sport Questionnaire (Appendix A, Table A1) and to analyze performance with motivational variables and adherence in young athletes in competitive sports.

The results of this study support a number of psychometric qualities of the scale, including its factorial composition, internal consistency, and test–retest reliability over a 30 days period. The predictive validity of the Perceived Performance in Sport Questionnaire was supported through the examination of the relations between perceived performance and the points scored. In this sense, the points scored have already been used in a previous study [48] with basketball players as an indicator of performance. Moreover, the results of the structural equation models show the predictive relationship between competence and perceived performance, as found by some previous studies [25,44]. Likewise, the intrinsic motivation predicted performance, coinciding with the findings of other studies [22,56].

The results of structural equation modelling show that the intention to be physically active was significantly predicted by the intrinsic motivation (direct and indirect effect) and the perceived performance. The relationship between intrinsic motivation and the intention to be physically active has already been shown in other studies [33,56,57]. On the other hand, perceived performance predicted the intention of young athletes to remain active. This relationship may be due to the fact that the athlete’s perceived performance can affect their self-esteem, a variable that has already been related to the intention to continue practicing sports [58].

For the first part of the structural equation model, we expected to find that the satisfaction of the three basic psychological needs predicted intrinsic motivation, as other studies did [59,60]. However, it was only the need for autonomy and relatedness that predicted intrinsic motivation in a positive and statistically significant way. In another study with Mexican soccer players [61], satisfaction of the need for competition was not shown to be a positive predictor of autonomous motivation (although, as in our study, they did present positive correlations). Future studies will have to study the reason for this lack of predictive power of competence on the intrinsic motivation of athletes. For the relationship between the need for competence and perceived performance, it was found that competence positively predicted perceived performance. Along these lines, some previous studies [25,62] have highlighted the importance of providing competence support to improve team performance.

On the other hand, it is interesting to highlight the importance of the athlete having an intrinsic motivation towards the practice of their sport, since the intrinsic motivation predicted the intention to be physically active and the perceived performance. In fact, some studies affirm that the intrinsic motivation [62] or autonomous motivation [14] of athletes must be increased to achieve success in sport. Furthermore, intrinsic motivation in young athletes is a predictor of their adherence to sports practice [33,57,60]. In this sense, research in the sports context has shown that autonomous motivation [33,63] has more positive consequences than controlled motivation [64,65] and, of course, than amotivation [41].

An interesting and original result of this study is that the athlete’s perceived performance predicted their intention to remain physically active in the future. In turn, this study shows that the satisfaction of the need for competence positively affects perceived performance, so employing strategies in training and in competition so that the athlete feels competent and so that coaches provide adequate feedback, recognizing effort and self-improvement, help them to set realistic sports goals or objectives, etc. [34]. This can help improve the perception of their performance and their intention to continue practicing this sport.

Although the satisfaction of basic psychological needs, experienced motivation or perceived performance are factors that determine adherence to sport, there are other variables such as enjoyment [66], implicit beliefs in their ability [67], and family or social support [68,69] that also determine the intention to continue practicing sport, so they should be considered jointly in future studies.

This study provides psychometric support for the Perceived Performance in Sport Questionnaire. Nevertheless, as for any research, some limitations need to be considered. First, this study was carried out with samples of young Spanish athletes; in the future, other similar groups of athletes should be examined. Second, the level of measurement invariance has not been studied (dependance on the gender or age of the athletes). Moreover, the different training backgrounds as well as the prevalence of team sports practitioners among the recruited players could be another limiting factor to be considered in future studies. In addition, the validation of an instrument must be treated as a continuous process, so the Perceived Performance in Sports Questionnaire will have to be tested again with athletes at a different competitive level, from different sports and from different areas of Spain. Likewise, it is recommended that future research continues to study the relationship between basic psychological needs, motivation and other variables with performance and adherence to sport, using other research designs (quasi-experimental, longitudinal studies, etc.).

## 6. Conclusions

In conclusion, this study showed that the satisfaction of the needs for autonomy and relatedness positively predicted intrinsic motivation. Satisfaction of the need for competence and intrinsic motivation predicted the athlete’s perceived performance. For its part, intrinsic motivation and perceived performance predicted the athlete’s intention to be physically active in the future. Furthermore, the evidence presented here supports the reliability and validity of the Perceived Performance in Sport Questionnaire (Appendix A, Table A1), and we hope that others will use this measure to further advance knowledge of athlete behavior and improve the training process.

## Figures and Tables

**Table 1:** Exploratory factor analysis.

Items	Factor Loading
I consider my performance is being good.	0.82
I am satisfied with my results in the competition.	0.67
I feel that I am collaborating with my effort and my performances in competition to improve the competitive level of the club or the team	0.62
I feel like I’m doing very well in the competition	0.84
I am offering good performance	0.69
**Explained variance**	62.3%
**Eigenvalue**	3.12

**Table 2:** Descriptive statistics and bivariate correlations.

	*M*	*SD*	1	2	3
1. Perceived performance	3.80	0.69	-	0.53 **	0.21
2. Points scored	5.94	4.20	-	-	0.60 **
3. Playing time	21.09	5.67	-	-	-

Notes: *M* = mean. *SD* = standard deviation. ** *p* < 0.001.

**Table 3:** Descriptive statistics and bivariate correlations.

	*M*	*SD*	1	2	3	4	5	6
1. Competence	4.48	0.51	-	0.48 **	0.43 **	0.24 **	0.51 **	0.55 **
2. Autonomy	4.05	0.63	-	-	0.37 **	0.25 **	0.39 **	0.30 **
3. Relatedness	4.74	0.47	-	-	-	0.32 **	0.32 **	0.48 **
4. Intrinsic motivation	6.44	0.61	-	-	-	-	0.35 **	0.30 **
5. Perceived performance	4.10	0.73	-	-	-	-	-	0.33 **
6. Intention to be physically active	4.74	0.44	-	-	-	-	-	-

Notes: *M* = mean. *SD* = standard deviation. ** *p* < 0.001.

## Appendix A. Perceived Performance in Sport Questionnaire

**Table A1:** This questionnaire was validated in Spanish.

Overall, during the Competition:	En General, Durante la Competición
1. I believe my performance is good.	1. Considero que mi rendimiento está siendo bueno
2. I am satisfied with my results in the competition.	2. Estoy satisfecho con mis resultados en la competición
3. I feel that I am helping, through my effort and my performance in competition, to improve the competitive level of the club or the team	3. Siento que estoy colaborando, con mi esfuerzo y mis actuaciones en competición, a mejorar el nivel competitivo del club o del equipo
4. I feel that I am performing very well in the competition	4. Siento que lo estoy haciendo muy bien en la competición
5. I am providing a good performance	5. Estoy ofreciendo un buen rendimiento

## References

[1] Soares A., Leonardi T.J., Silva J., Nascimento J.V., Paes R.R., Goncalves C.E., Carvalho H.M. (2020). Performance, motivation, and enjoyment in young female basketball players: An interdisciplinary approach. J. Sports Sci..

[2] Arribas-Galarraga S., Saies E., Cecchini J.A., Arruza J.A., Luis-De-Cos I. (2017). The relationship between emotional intelligence, self-determined motivation and performance in canoeists. J. Hum. Sport Exerc..

[3] Ruiz-Vanoye J.A., Díaz-Parra O., Fuentes-Penna A., Vélez-Díaz D., García-Munguía M., Ruiz-Díaz J., Ruiz-Díaz F. (2017). Motivation Index to Improve the Soccer Performance. Int. J. Comb. Optim. Probl. Inform..

[4] Abdullah M.R., Musa R.M., Maliki A.B.H.M.B., Kosni N.A., Suppiah P.K. (2016). Role of psychological factors on the performance of elite soccer players. J. Phys. Educ. Sport.

[5] Olmedilla A., Ortega E., Andreu M.D., Ortín F.J. (2010). Programa de intervención psicológica en futbolistas: Evaluación de habilidades psicológicas mediante el CPRD [A psychological intervention programme for footballers: An evaluation of psychological skills through the CPRD]. Rev. Psicol. Deport..

[6] Anderson R., Hanrahan S.J., Mallett C.J. (2014). Investigating the Optimal Psychological State for Peak Performance in Australian Elite Athletes. J. Appl. Sport Psychol..

[7] Arthur R.S., Fitzwater J., Roberts R., Hardy J., Arthur C.A. (2017). Psychological Skills and “the Paras”: The Indirect Effects of Psychological Skills on Endurance. J. Appl. Sport Psychol..

[8] Olmedilla A., Torres-Luque G., García-Mas A., Rubio V.J., Ducoing E., Ortega E. (2018). Psychological profiling of triathlon and road cycling athletes. Front. Psychol..

[9] Formenti D., Duca M., Trecroci A., Ansaldi L., Bonfanti L., Alberti G., Iodice P. (2019). Perceptual vision training in non-sport-specific context: Effect on performance skills and cognition in young females. Sci. Rep..

[10] Trecroci A., Boccolini G., Duca M., Formenti D., Alberti G. (2020). Mental fatigue impairs physical activity, technical and decision-making performance during small-sided games. PLoS ONE.

[11] Gimeno F., Buceta J.M., Pérez-Llantada M.C. (2007). Influencia de las variables psicológicas en el deporte de competición: Evaluación mediante el cuestionario Características psicológicas relacionadas con el rendimiento deportivo [The influence of psychological variables on sports performance: Assessment w. Psicothema.

[12] Claver F., Jiménez R., Conejero M., García-González L., Moreno M.P. (2015). Cognitive and motivational variables as predictors of performance in game actions in young volleyball players. Eur. J. Hum. Mov..

[13] Fletcher D., Sarkar M. (2012). A grounded theory of psychological resilience in Olympic champions. Psychol. Sport Exerc..

[14] Gillet N., Vallerand R.J. (2016). Les effets de la motivation sur la performance sportive au regard de la théorie de l’autodétermination: Vers une approche intra-individuelle. Psychol. Fr..

[15] Gómez-López M., Granero-Gallegos A., Isorna-Folgar M. (2013). Análisis de los factores psicológicos que afectan a los piragüistas en el alto rendimiento [Analysis of psychological factors that affect high athletic performance in kayakers]. Rev. Iberoam. Diagn. Eval..

[16] MacNamara Á., Button A., Collins D. (2010). The role of psychological characteristics in facilitating the pathway to elite performance part 2: Examining environmental and stage-related differences in skills and behaviors. Sport Psychol..

[17] Ryan R.M., Deci E.L. (2000). Self-Determination Theory and the facilitation of intrinsic motivation, social development, and well-being. Am. Psychol..

[18] Deci E.L., Ryan R.M. (1985). Intrinsic Motivation and Self-Determination in Human Behavior.

[19] Lonsdale C., Hodge K., Rose E.A. (2008). The Behavioral Regulation in Sport Questionnaire (BRSQ): Instrument development and initial validity evidence. J. Sport Exerc. Psychol..

[20] Ryan R.M., Deci E.L. (2017). Self-Determination Theory. Basic Psychological Needs in Motivation.

[21] Gillet N., Vallerand R.J., Rosnet E. (2009). Motivational clusters and performance in a real-life setting. Motiv. Emot..

[22] Gillet N., Berjot S., Vallerand R.J., Amoura S., Rosnet E. (2012). Examining the motivation-performance relationship in competitive sport: A cluster-analytic approach. Int. J. Sport Psychol..

[23] Pope J.P., Wilson P.M. (2015). Testing a sequence of relationships from interpersonal coaching styles to rugby performance, guided by the coach-athlete motivation model. Int. J. Sport Exerc. Psychol..

[24] Ryan R.M., Deci E.L. (2020). Intrinsic and extrinsic motivation from a self-determination theory perspective: Definitions, theory, practices, and future directions. Contemp. Educ. Psychol..

[25] Cerasoli C.P., Nicklin J.M., Nassrelgrgawi A.S. (2016). Performance, incentives, and needs for autonomy, competence, and relatedness: A meta-analysis. Motiv. Emot..

[26] Sheldon K.M., Zhaoyang R., Williams M.J. (2013). Psychological need-satisfaction, and basketball performance. Psychol. Sport Exerc..

[27] Moreno J.A., Moreno R., Cervelló E. (2007). El autoconcepto físico como predictor de la intención de ser físicamente activo [The physical self-concept as predictor of the intention of being physically active]. Pysicol. Salud.

[28] Lindwall M., Hassmén P. (2004). The role of exercise and gender for physical self-perceptions and importance ratings in Swedish university students. Scand. J. Med. Sci. Sport.

[29] Wankel L.M., Berger B.G. (1990). The psychological and social benefits of sport and physical activity. J. Leis. Res..

[30] Eime R.M., Young J.A., Harvey J.T., Charity M.J., Payne W.R. (2013). A systematic review of the psychological and social benefits of participation in sport for adults: Informing development of a conceptual model of health through sport. Int. J. Behav. Nutr. Phys. Act..

[31] Goudas M., Biddle S.J.H., Underwood M. (1995). It ain’t what you do, It’s the Way That You Do It! Teaching Style Affects Children’s Motivation in Track and Field Lessons. Sport Psychol..

[32] Vallerand R.J., Roberts G.C. (2001). A hierarchical model of intrinsic and extrinsic motivation in sport and exercise. Advances in Motivation in Sport and Exercise.

[33] Almagro B.J., Sáenz-López P., Moreno J.A. (2010). Prediction of sport adherence through the influence of autonomy-supportive coaching among Spanish adolescent athletes. J. Sport. Sci. Med..

[34] Almagro B.J., Sáenz-López P., Moreno-Murcia J.A., Spray C. (2015). Motivational factors in young Spanish athletes: A qualitative focus drawing from self-determination theory and achievement goal perspectives. Sport Psychol..

[35] Crane J., Temple V. (2015). A systematic review of dropout from organized sport among children and youth. Eur. Phys. Educ. Rev..

[36] Fraser-Thomas J., Côté J., Deakin J. (2008). Understanding dropout and prolonged engagement in adolescent competitive sport. Psychol. Sport Exerc..

[37] Cervelló E.M., Escartí A., Guzmán J.F. (2007). Youth sport dropout from the achievement goal theory. Psicothema.

[38] Molinero O., Salguero A., Tuero C., Alvarez E., Márquez S. (2006). Dropout reasons in young Spanish athletes: Relationship to gender, type of sport and level of competition. J. Sport Behav..

[39] Oliveira G., Araújo I., Junior O.A., Neto J.B., Cielo F.L. (2007). A Relação entre a Especialização Precoce e o Abandono Prematuro da Natação. Mov. Percepção.

[40] Pineda-Espejel H.A., López-Walle J.M., Tomás I. (2017). Influencia del entrenador deportivo con relación al perfeccionismo y las orientaciones de meta [Influence of the sports coach in relation to perfectionism and goal orientations]. Rev. Psicol. Deport..

[41] Pulido J.J., Sánchez-Oliva D., Sánchez-Miguel P.A., Amado D., García-Calvo T. (2018). Sport commitment in young soccer players: A self-determination perspective. Int. J. Sport. Sci. Coach..

[42] Mageau G.A., Vallerand R.J. (2003). The coach-athlete relationship: A motivational model. J. Sports Sci..

[43] Ajzen I., Fishbein M., Lohmann S., Albarracin D., Albarracín D., Johnson B.T., Zanna M.P. (2005). The Influence of Attitudes on Behavior. The Handbook of Attitudes.

[44] Lourenço J., Almagro B.J., Sáenz-López P. (2018). Validação do Questionário de Perceção do Rendimento no Desporto (QPRD) [Validation of the Perceived Performance in Sport Questionnaire]. J. Sport Sci..

[45] American Psychological Association (2010). Publication Manual of American Psychological Association.

[46] Caparrós-Pons T., Padulés-Riu J.M., Rodas-Font G., Capdevila L. (2014). ¿La fuerza puede predecir el rendimiento y la lesionabilidad en el baloncesto profesional? [Can the Strenght Predict the Performance and Injury rates in Professional Basketball?]. Apunt. Educ. Fis. Deport..

[47] Ortega-Toro E., Bernal-Polo J., Gómez-Ruano M.A., Giménez-Egido J.M., Verdú-Conesa I. (2019). Relación entre edad y criterios de rendimiento y participación en jugadores de baloncesto de alto rendimiento [Relationship between age and performance and participation in high performance basketball players]. Rev. Psicol. Deport..

[48] León-Prados J.A., Fuentes I., Calvo A. (2014). Relationship between anxiety state, self-confidence and performance in basketball. Rev. Int. Med. Cienc. Act. Fis. Deport..

[49] Alfonso J.D., Ortega E., Palao J.M. (2009). Age, participation time and performance in basketball players in olympic games. Rev. Psicol. Deport..

[50] Hu L.T., Bentler P.M. (1999). Cutoff criteria for fit indexes in covariance structure analysis: Conventional criteria versus new alternatives. Struct. Equ. Model..

[51] Sánchez J.M., Núñez J.L. (2007). Análisis preliminar de las propiedades psicométricas de la versión española de la Escala de Necesidades Psicológicas Básicas en el Ejercicio Físico. Rev. Iberoam. Psicol. Ejerc. Deport..

[52] Vlachopoulos S.P., Michailidou S. (2006). Development and initial validation of a measure of autonomy, competence, and relatedness: The Basic Psychological Needs in Exercise Scale. Meas. Phys. Educ. Exerc. Sci..

[53] Almagro B.J., Sáenz-López P., Moreno-Murcia J.A. (2012). Perfiles motivacionales de deportistas adolescentes españoles [Motivational profiles of Spanish adolescent athletes]. Rev. Psicol. Deport..

[54] Viladrich C., Torregrosa M., Cruz J. (2011). Calidad psicométrica de la adaptación española del cuestionario de regulación conductual en el deporte [Psychometric quality supporting the Spanish adaptation of the Behavioral Regulation in Sport Questionnaire]. Psicothema.

[55] Hein V., Müür M., Koka A. (2004). Intention to be physically active after school graduation and its relationship to three types of intrinsic motivation. Eur. Phys. Educ. Rev..

[56] Cerasoli C.P., Nicklin J.M., Ford M.T. (2014). Intrinsic Motivation and Extrinsic Incentives Jointly Predict Performance: A 40-year meta-analysis. Psychol. Bull..

[57] Almagro B.J., Conde C. (2012). Factores motivacionales como predictores de la intención de ser físicamente activos en jóvenes jugadores de baloncesto [Motivational factors as predictors of young basketball players´ intention to be physically active]. Cuad. Psicol. Deport..

[58] Franco E., Coteron J., Gomez V. (2017). Promotion of Physical Activity in Adolescents: Role of Motivation and Self-esteem. Psiencia-Rev. Latinoam. Cienc. Psicol..

[59] Almagro B.J., Sáenz-López P., González-Cutre D., Moreno-Murcia J.A. (2011). Clima motivacional percibido, necesidades psicológicas y motivación intrínseca como predictores del compromiso deportivo en adolescentes [Perceived motivational climate, psychological needs and intrinsic motivation as Predictors of sport commitment in ado. Rev. Int. Cienc. Deporte.

[60] Jõesaar H., Hein V., Hagger M.S. (2011). Peer influence on young athletes’ need satisfaction, intrinsic motivation and persistence in sport: A 12-month prospective study. Psychol. Sport Exerc..

[61] Cantú-Berrueto A., Castillo I., López-Walle J., Tristán J., Balaguer I. (2016). Coach interpersonal style, basic psychological needs and motivation: A study in Mexican college football players. Rev. Iberoam. Psicol. Ejerc. Deport..

[62] Mertens N., Boen F., Vande Broek G., Vansteenkiste M., Fransen K. (2018). An experiment on the impact of coaches’ and athlete leaders’ competence support on athletes’ motivation and performance. Scand. J. Med. Sci. Sport..

[63] Monteiro D., Teixeira D.S., Travassos B., Duarte-Mendes P., Moutão J., Machado S., Cid L. (2018). Perceived effort in football athletes: The role of achievement goal theory and self-determination theory. Front. Psychol..

[64] Ntoumanis N., Standage M. (2009). Morality in sport: A self-determination theory perspective. J. Appl. Sport Psychol..

[65] Ramis Y., Torregrosa M., Viladrich C., Cruz J. (2017). The effect of coaches’ controlling style on the competitive anxiety of young athletes. Front. Psychol..

[66] Gadner L.A., Magee C.A., Vella S.A. (2017). Enjoyment and Behavioral Intention Predict Organized Youth Sport Participation and Dropout. J. Phys. Act. Health.

[67] Gómez-López M., Merino-Barrero J.A., Manzano-Sánchez D., Valero-Valenzuela A. (2019). A cluster analysis of high-performance handball players’ perceived motivational climate: Implications on motivation, implicit beliefs of ability and intention to be physically active. Int. J. Sports Sci. Coach..

[68] Almagro B.J., Paramio-Pérez G. (2017). Motivación y adherencia a la práctica de baloncesto en adolescentes [Motivation and adherence to practice of basketball in adolescent]. Cuad. Psicol. Deport..

[69] Lavallee D., Sheridan D., Coffee P., Daly P. (2019). Psychosocial Intervention A Social Support Intervention to Reduce Intentions to Drop-out from Youth. Psychosoc. Interv..

